# SPECT/CT in the Diagnosis of Ectopic Gastric Mucosa-Meckel's Diverticulum

**DOI:** 10.1055/s-0044-1787719

**Published:** 2024-06-11

**Authors:** Zehra Pınar Koç, Pınar Pelin Özcan, Ferah Tuncel, Caner İsbir, Yusuf Usta

**Affiliations:** 1 Department of Nuclear Medicine, Mersin University, Mersin, Türkiye; 2 Department of Pathology, Mersin University, Mersin, Türkiye; 3 Department of Pediatric Surgery, Mersin University, Mersin, Türkiye; 4 Department of Pediatric Gastroenterology, Mersin University, Mersin, Türkiye

**Keywords:** Meckel's diverticulum, scintigraphy, SPECT/CT, ectopic gastric mucosa

## Abstract

**Aim** The imaging of Meckel's diverticulum (MD) is based of accumulation of Tc-99m pertechnetate in the ectopic gastric mucosa (EGM) content. Although the diagnostic accuracy of this imaging modality is high, there are some overlap patients with coexisting gastrointestinal bleeding and false positive causes hampering diagnostic power. The aim of this study was to evaluate the possible contribution of single-photon emission computed tomography/computed tomography (SPECT/CT) in EGM-MD diagnosis and to determine the indication of this additional imaging modality.

**Materials and Methods** Fifty-two pediatric patients (24 girls, 28 boys; mean age: 8.06 ± 5.22 years old) who have suspicion of MD and referred for scintigraphy were evaluated retrospectively. Additional SPECT/CT were performed to selected five cases among the group. The results of the scintigraphy as well as SPECT/CT were compared with endoscopy, pathology, and/or follow-up results.

**Results** There were 9 patients with equivocal study results, 12 positive results, and the others were considered negative MD scintigraphy. One patient was out of follow-up and 10 patients underwent surgery. Only one single patient was negative during surgery but scintigraphy was also negative. The diagnostic sensitivity, specificity, and accuracy were 100, 95, and 96%, respectively. Among five patients with SPECT/CT results one patient was diagnosed by only SPECT/CT who had EGM in duplication cyst, one equivocal patient was diagnosed as descending colon bleeding, and one patient's lesion was clearly delineated by SPECT/CT.

**Conclusion** SPECT/CT has clear advantage over standard planar scintigraphy imaging in EGM-MD determination. This modality might decrease equivocal and false positive results but this issue has to be addressed with further studies.

## Introduction

The frequency of Meckel's diverticulum (MD) in normal population is 0.14 to 4.5% 1 2 and rarely presents with complications (4.2–6.4%). 3 Due to the ectopic gastric mucosa (EGM) content which might be present in nearly half of the patients, first presentation can be gastrointestinal bleeding. Scintigraphy determines the EGM content of the MD thus depicts surgical resection candidates and prevents from unnecessary surgery. The diagnostic accuracy of the modality is considerably high with sensitivity and specificity of 80 to 90% and 95%, respectively, for children. 4 However, the diagnostic efficiency is hampered as the age of the patient increases and significantly lower for the adults. 4 The possible contribution of the single-photon emission computed tomography/computed tomography (SPECT/CT) to the diagnostic workup of MD scintigraphy was not sufficiently considered previously in the literature. This may be due to high diagnostic efficiency of the modality and because the SPECT/CT is not available in every nuclear medicine department. There are case reports and a case series in the literature which address significant impact on diagnostic confidence. 1 5 6 7 8 The aim of this study was to analyze the possible effect of SPECT/CT in the diagnostic efficiency of the MD scintigraphy to determine EGM.

## Materials and Methods

The patients (24 girls, 28 boys; mean age: 8.06 ± 5.22 years old) who were referred to the nuclear medicine department for MD-EGM scintigraphy were retrospectively analyzed. The patients were all at pediatric age (< 18 years) but equally distributed age population including adolescent patients.

### Scintigraphy and SPECT/CT Imaging

The informed consents of the patients' guardians were obtained prior to imaging procedure. The imaging was performed with prior medication of proton-pomp inhibitors as a premedication. No prior starvation was suggested. The radiopharmaceutical was injected via venous line at a dose of approximately 0 mCi (37 mBq/adjusted according to the body weight). Dynamic imaging was performed with following sequential planar anteroposterior and lateral spot imaging and additional SPECT/CT were performed just after the planar imaging procedure in case of undetermined results in planar imaging in five cases. Two different scanners (Symbia, Siemens SPECT gamma camera and positron emission tomography [PET]-CT scanners Siemens MCT 20, respectively) were used for the fusion SPECT/CT imaging. The fusion analysis was performed with the MCT20 PET-CT scanners imaging console and reevaluated by an experienced nuclear medicine physician at Mc-OsiriX reading console. Focal significant increased activity in the abdominal region with simultaneous gastric uptake was considered positive for MD. Additional erythrocyte-labeled scintigraphy was performed in one case with inconclusive results in MD scintigraphy by administration of Tc-99m pertechnetate adjusted according to the pediatric dosage chart to the patient via direct intravenous administration of the radiopharmaceutical and about 1 hour later direct intravenous administration of the pyrophosphate on the other arm. The imaging was performed by the same methodology and equipment described above. The dose for additional CT imaging was adjusted according to the body weight by the automatic program of the PET/CT scanner (smart mA). The imaging results of the patients were compared with the patient's endoscopy, follow-up, and pathology surgery results and the diagnostic sensitivity, specificity, and accuracy of the study were obtained.

## Results

The imaging results were considered positive, negative, and equivocal regarding the diagnosis of EGM-MD. In case of equivocal results, the erythrocyte-labeled scintigraphy for bleeding site determination was performed in selected cases. The equivocal results were considerably high in the patient group ( *n* = 10). Nine patients were determined as MD positive and others were negative. All the nine cases were verified by surgery pathology results. Only single patient's surgery results were negative who had negative scintigraphy results also.

**Fig. 1:**
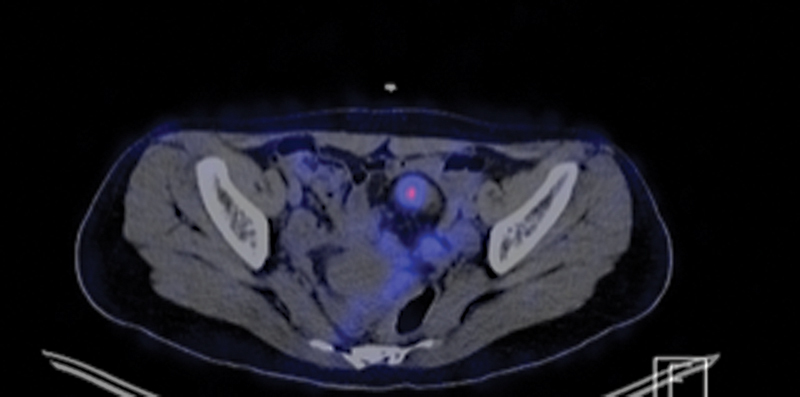
SPECT-CT images of a 15 year old adolescent girl with verified Ectopic Gastric Mocase at a Dublication cyst as determined by Meckel's Diverticulum Scintigraphy.

**Fig. 2:**
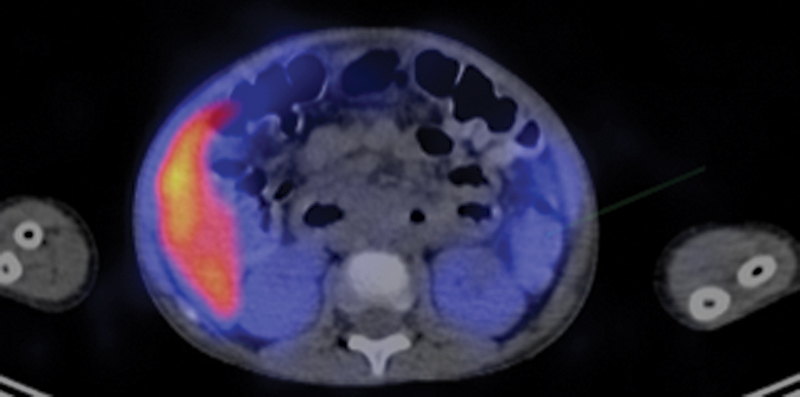
Single-photon emission computed tomography/computed tomography (SPECT/CT) images of a 13-year-old female patient demonstrating focal uptake of Tc-99m pertechnetate in the bowel at left midline with pathological diagnosis of Meckel's diverticulum and ectopic gastric mucosa.

**Fig. 3:**
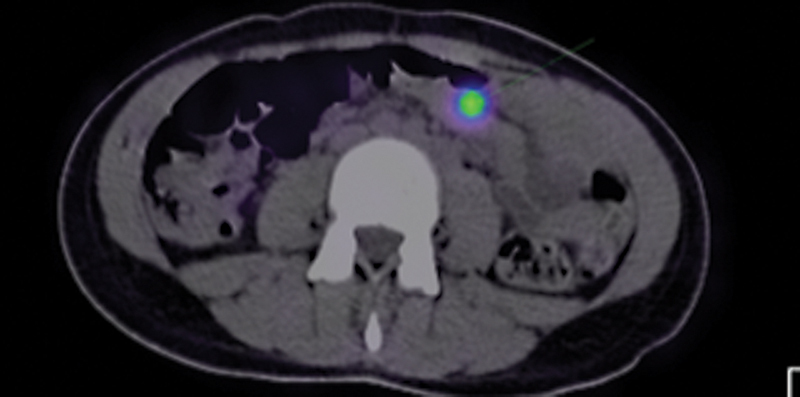
Erythrocyte-labeled scintigraphy—single-photon emission computed tomography/computed tomography (SPECT/CT) images of a patient whose Meckel's diverticulum scintigraphy pointed out gastrointestinal bleeding rather than ectopic gastric mucosa (EGM) and finally the bleeding site was determined only by SPECT/CT at splenic flexure adjacent to spleen activity.

There were five patients with additional SPECT/CT imaging. Among these patients EGM were determined in two. One of the patients' EGM in a duplication cyst was diagnosed only by SPECT/CT whose planar imaging was considered equivocal ( Fig. 1 ). Another patient with equivocal findings, bleeding site was determined by SPECT/CT ( Fig. 2 ). Clear anatomic depiction of one of the patients was provided by SPECT/CT ( Fig. 3 ). Diagnostic sensitivity, specificity, and accuracy of the imaging modality were 100, 95, and 96%, respectively.

## Discussion

The determination of the MD-EGM by means of MD scintigraphy is possible with high diagnostic accuracy according to the study results. However, there are some considerable numbers of equivocal cases and overlap patients with gastrointestinal bleeding. This may be due to the age of the study group which consists of adolescent patients as well. Previous literature data suggest that the diagnostic accuracy of the MD scintigraphy decreases as the age of the patients increases and the diagnostic efficacy of the MD scintigraphy is considerably low in adults. 4 In case of these situations, SPECT/CT imaging provides true positive results. The patient with an EGM in the duplication cyst would be the only false negative case in the study group if we did not perform additional SPECT/CT. EGM of tubular intestinal duplication was also determined by SPECT/CT in another patient in the literature previously. 9

MD scintigraphy usually is considered sufficient for diagnosis of EGM in MD. However, there are false positive and false negative results as the patients' age increase. Additionally, in the problematic cases with previous abdominal surgery history of SPECT/CT with additional radioguided surgery provided promising results. 10 11

Previous data also suggested that the contribution of anatomical detail information increases the surgical success. 12

There is a considerable number of false positives in the MD imaging especially due to the excretion of the radiopharmaceutical through urine. The possible intervention to exclude this false positivity is to obtain lateral spot imaging which indicate activity in the posterior aspect of the verifying urinary tract. However, there are specific exceptions that this intervention could not benefit. In a previous case series one of the patients with pelvic ectopic kidney was only determined by SPECT/CT. 8

SPECT/CT provided clear delineation of the EGM in another patients' MD. Another patient who was considered equivocal was also determined as gastrointestinal bleeding by SPECT/CT. These encouraging results showed that SPECT/CT might contribute to diagnostic accuracy of MD in case it is performed in special cases with equivocal results. There are previous case report and review about the contribution of SPECT/CT in the diagnosis of bleeding site as an adjunct with MD scintigraphy. 13 14 Especially in the adolescents or adults the possible diagnosis of non-MD bleeding and additional bleeding scintigraphy with SPECT/CT should be considered. The equivocal results in our series usually belong to the adolescent patients. This issue should be considered as the age increases the results of the MD scintigraphy might be challenging. A recent case report verified the role of SPECT/CT in a 17-year-old boy with atypical presentation of MD which was false negative with only scintigraphy imaging. 5 Similarly, in case of complicated MD the diagnostic interpretation might be challenging and SPECT/CT could demonstrate which was shown in a report of a 15-year-old boy. 6 Adjacent anatomic structures including a diverticulum might also complicate diagnosis. 15 Previous studies have shown that false negativity might be a significant problem as well as false positivity. 16 17 SPECT/CT might also contribute in these problems.

The limitation of this report is the small number of patients with SPECT/CT results which can be explained by the fact that additional CT dose concern to the children. The SPECT/CT should be preserved for the selected patients with equivocal results. Although the dose of CT is usually adjusted in pediatric patients in most of the centers, the dose consideration is the most important problem against these kinds of studies. However, in case of missed diagnosis possible complications and unnecessary surgical interventions should be considered for decision also. The SPECT/CT should be preferred for the selected patients with equivocal results.

## Conclusion

The MD scintigraphy is a highly accurate modality in the determination of EGM but SPECT/CT might be considered in special circumstances especially in equivocal scintigraphy results in adolescent cases. This approach might decrease false positive and false negative interpretations.
